# Mapping a mammalian adult adrenal gland hierarchy across species by microwell-seq

**DOI:** 10.1186/s13619-020-00042-8

**Published:** 2020-08-03

**Authors:** Shujing Lai, Lifeng Ma, Weigao E, Fang Ye, Haide Chen, Xiaoping Han, Guoji Guo

**Affiliations:** 1 Center for Stem Cell and Regenerative Medicine, Zhejiang University School of Medicine, Hangzhou, 310058 China; 2 Bone Marrow Transplantation Center, The First Affiliated Hospital, Zhejiang University School of Medicine, Hangzhou, 310009 China; 3 Institute of Hematology, Zhejiang University, Hangzhou, 310058 China

## Abstract

Recently, single-cell RNA-seq technologies have been rapidly updated, leading to a revolution in biology. We previously developed Microwell-seq, a cost-effective and high-throughput single cell RNA sequencing(scRNA-seq) method with a very simple device. Most cDNA libraries are sequenced using an expensive Illumina platform. Here, we present the first report showing combined Microwell-seq and BGI MGISEQ2000, a less expensive sequencing platform, to profile the whole transcriptome of 11,883 individual mouse adult adrenal gland cells and identify 18 transcriptionally distinct clusters. Moreover, we performed a single-cell comparative analysis of human and mouse adult adrenal glands to reveal the conserved genetic networks in these mammalian systems. These results provide new insights into the sophisticated adrenal gland hierarchy and provide a benchmark, low-cost strategy for high-throughput single-cell RNA study.

## Background

Cells are the basic unit of life, and cells within a tissue exhibit high heterogeneity. Single-cell RNA-sequencing (scRNA-seq) has become a benchmark method for dissecting cell heterogeneity, unraveling cell status, and identifying cell types (Hashimshony et al., 2012; Ramskold et al., 2012; Treutlein et al., 2014; Shalek et al., 2013; Tang et al., 2009). The cost of single-cell sequencing is mainly based on library construction and sequencing. Recently, massive, parallel assays can process thousands of single cells simultaneously for the assessment of their transcriptional profiles at rapidly decreasing library costs (Macosko et al., 2015; Klein et al., 2015; Cao et al., 2017; Gierahn et al., 2017). We previously developed Microwell-seq, a cost-effective and high-throughput scRNA-seq method with a very simple device, making the library-construction price less than 1 dollar per cell. Using Microwell-seq, we mapped the first mammalian cell atlas and revealed the evolutionary conservation of the hematopoietic hierarchy across species (Lai et al., 2018; Han et al., 2018).

Most cDNA libraries are sequenced using an expensive Illumina sequencing platform (Goodwin et al., 2016; Natarajan et al., 2019). BGI (Beijing Genomics Institute, China) developed an alternative combinatorial probe-anchor synthesis-based sequencing platform, BGISEQ500, in 2015, which has been applied to small noncoding RNA sequencing, ancient DNA sequencing for paleogenomic analysis, human genome resequencing and scRNA sequencing (Fehlmann et al., 2016; Huang et al., 2018; Mak et al., 2018). Recently, BGI launched the less-expensive MGISEQ2000 sequencing platform as an alternative to Illumina HiSeq and BGISEQ500.

The adrenal gland sites near the upper part of the kidney play important roles in secreting hormones and adrenaline (Mihai, 2019). The adrenal gland tremendously impacts the functioning of all tissues, glands, and organs in the body (Ramlagun et al., 2018; Peng et al., 2019; Reincke et al., 2019; Soedarso et al., 2019). The previously published Mouse Cell Atlas does not cover adrenal gland data; therefore, we decided to map the mouse adrenal gland at single-cell resolution (Han et al., 2018).

In this study, the associated application of the BGI platform and Microwell-seq greatly reduced the cost of single-cell analysis. Using Microwell-seq, we analyzed mouse adrenal glands with more than 10,000 single-cell transcriptomic profiles and defined 18 cell types according to published pipelines (Macosko et al., 2015). In addition, we assessed the properties of the BGI MGISEQ2000 sequencing platform for scRNA-seq and compared it with the most widely used Illumina HiSeq sequencing platform using uniform single-cell data. Finally, we performed a comparative transcriptomic analysis of the human and mouse adrenal gland cell atlases at single-cell resolution, defining similar cell subpopulation pairs across species.

## Results

### Mapping a mouse adult adrenal gland hierarchy by microwell-seq

**Fig. 1:**
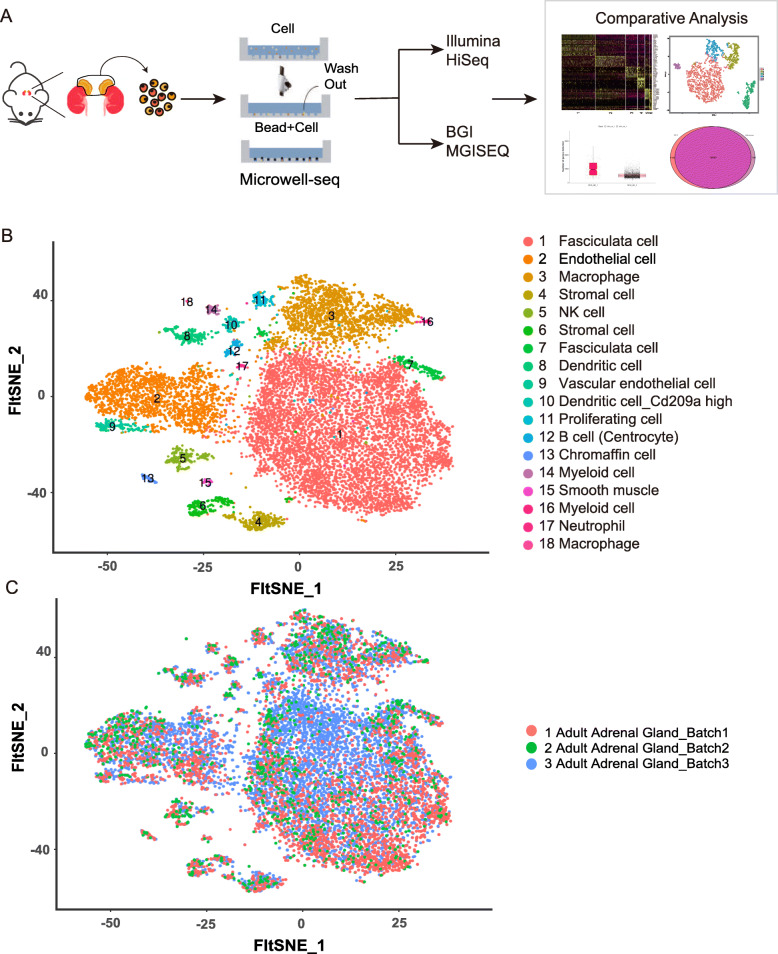
Mapping the mouse adult adrenal gland atlas using Microwell-seq (**a**) Descriptions of the experimental workflow of Microwell-seq and the comparison of two sequencing platforms. **b** A t-distributed stochastic neighbor embedding (t-SNE) map of 18 mouse adrenal gland clusters. Cells are colored by 1–18 cell-type clusters. **c** Three batches of 11,883 single mouse adrenal gland cells were visualized on a t-SNE map. Cells were labeled with three colors according to their batch source. Batches 1–3 belong to three mice

**Fig. 2:**
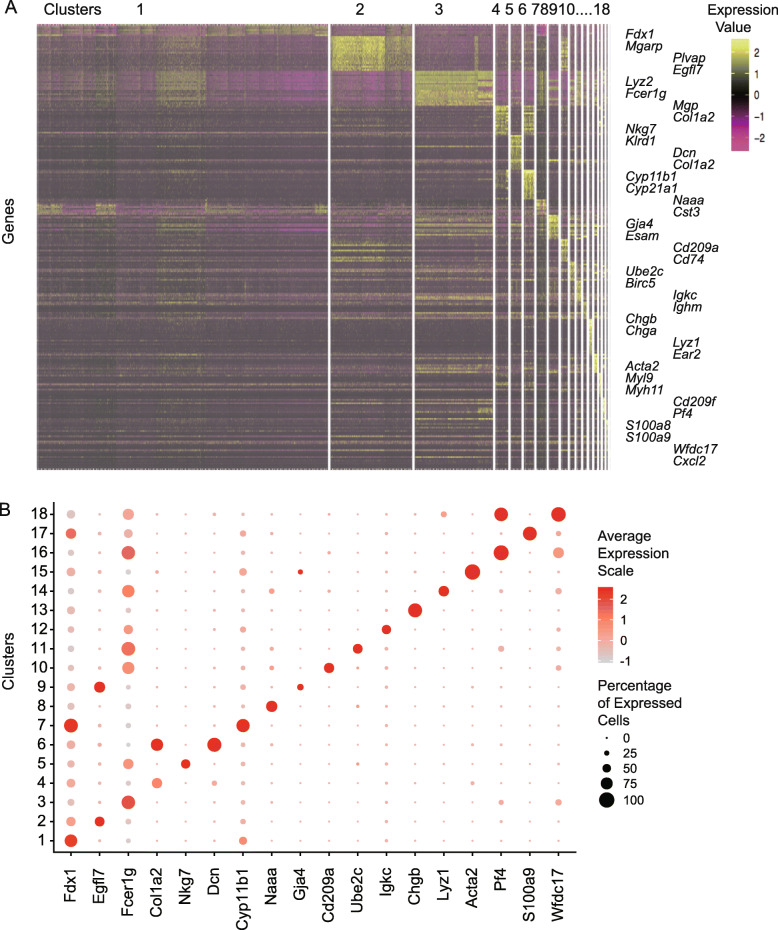
Gene expression pattern of the adult adrenal glands (**a**) Gene expression heat map showing the differentially expressed genes for each cell cluster in the mouse adrenal gland. Each point on the abscissa represents a cell, and each point on the ordinate represents a gene. The color bar on the right represents the Z-score. Yellow indicates high expression; purple and black indicate low expression. Representative genes are labeled in the corresponding area on the right side of the heat map. **b** Dot plots showing the representative gene expression for each cell cluster in the adult adrenal gland. The color encodes the average expression level. The color bar on the right represents the Z-score. Red indicates high expression; gray indicates low expression. The size of the dots denotes the percentage of a cell type

The whole workflow of our study is shown in Fig. 1a. Here, we used Microwell-seq to successfully profile the whole transcriptome of 11,883 individual mouse adrenal gland cells (Fig. 1b). Through bioinformatics analysis, we identified 18 transcriptionally distinct cell clusters (Fig. 1b). To decrease the cost of scRNA-seq, we used the BGI sequencing platform, which was presumed to be potentially cost-effective. Mouse adult adrenal gland cells from 3 independent Microwell-seq experiments mixed homogeneously on a t-SNE map (Fig. 1c). The gene expression levels of 11,883 mouse adrenal gland cells are shown in the heat map (Fig. 2a). The 18 cell clusters showed a highly specific gene expression pattern (Fig. 2b and Supplementary Figure 1). The defined cell type clusters and cluster-specific markers are summarized in Supplementary Table 1.

We found the presence of many cell types with interesting cluster-specific markers (Lai et al., 2018; Han et al., 2018). The significant expression of *Fdx1* and *Cyp11b1* suggested that C1 contained fasciculata cells (Bergman et al., 2017). In addition, *Cyp11a1, Meg3* and *Mgarp* were highly expressed in the C1 cells, supporting their fasciculata cell identity (Bergman et al., 2017). C2 cells were identified as endothelial cells because they expressed high levels of *Plvap, Egfl7, Kdr, Esam* and *Eng (Han et al.,*^2018^*).* Highly expressed markers, *Lyz2* and *Fcer1g,* defined C3 cells as macrophages. C4 was defined by stromal cells according to the *Mgp* gene and the gene expression of collagens such as *Col1a2* and *Col3a1* (Supplementary Figure 2). C5 was marked by the expression of *Klrd1, Nkg7, Klre1* and *Klrk1,* which suggested it contained natural killer cells (Bongen et al., 2018). Stromal cell-related genes, including *Dcn, Col1a2, Col1a1* and *Col3a1*, were also found in cluster C6. C7 cells showed fasciculata cell characteristics, with *Cyp11b1* highly expressed (Bergman et al., 2017). C8 cells showed high levels of genes such as *Naaa, Cst3, Irf8, Cd74, Plac8* and MHCII-related genes, indicating dendritic cell characteristics. C9 cells showed vascular endothelial cell characteristics, with highly expressed *Gja4, Esam* and *Egfl7 (Kim et al.,*^2019^*)*. Other vascular endothelial cell markers (*Cldn5*, *Eng* and *Cdh5*) were also expressed in the C9 cluster. C10 was defined by *Cd209a* high dendritic cell by marker genes, including *Cd209a, Cd74, H2-Ab1, H2-Eb1* and *H2-DMa.* C11 cells expressed high levels of proliferation marker genes, including *Ube2c, Birc5, Cdc20, C1qc, Ctsc, C1qb, Ctss* and *C1qa.* C12 was identified by the high expression of *Igkc, Cd79b* and *Ighm,* which suggests it contained B cells. C13 cells highly expressed chromaffin cell marker genes, including *Chgb, Chga, Npy, Scg2* and *Dlk1.* C14 was defined by myeloid cells because of the expression of *Lyz1, Ear2, Fn1, Lyz2* and *H2-DMb1.* C15 cells showed high levels of known smooth muscle markers, namely *Acta2*, *Myl9*, *Tpm2, Myh11, Mylk* and *Tpm1 (Regalado et al.,*^2018^*).* In addition, the C16 cells highly expressed myeloid markers, including *Cd209f, Pf4, Cd209d, Lyv1* and *Cd209b,* which differed from those expressed in C14 (Supplementary Figure 3). C17 was identified by the high expression of *S100a9* and *S100a8,* which suggests it contained neutrophils (Lai et al., 2018). Notably, markers of macrophages, including *Wfdc17*, *Cxcl2*, *Lyz2* and *Cd14,* were highly expressed in cluster C18, which was defined by macrophages.

### Comprehensive comparison of the BGI MGISEQ2000 and Illumina HiSeq platforms

**Fig. 3:**
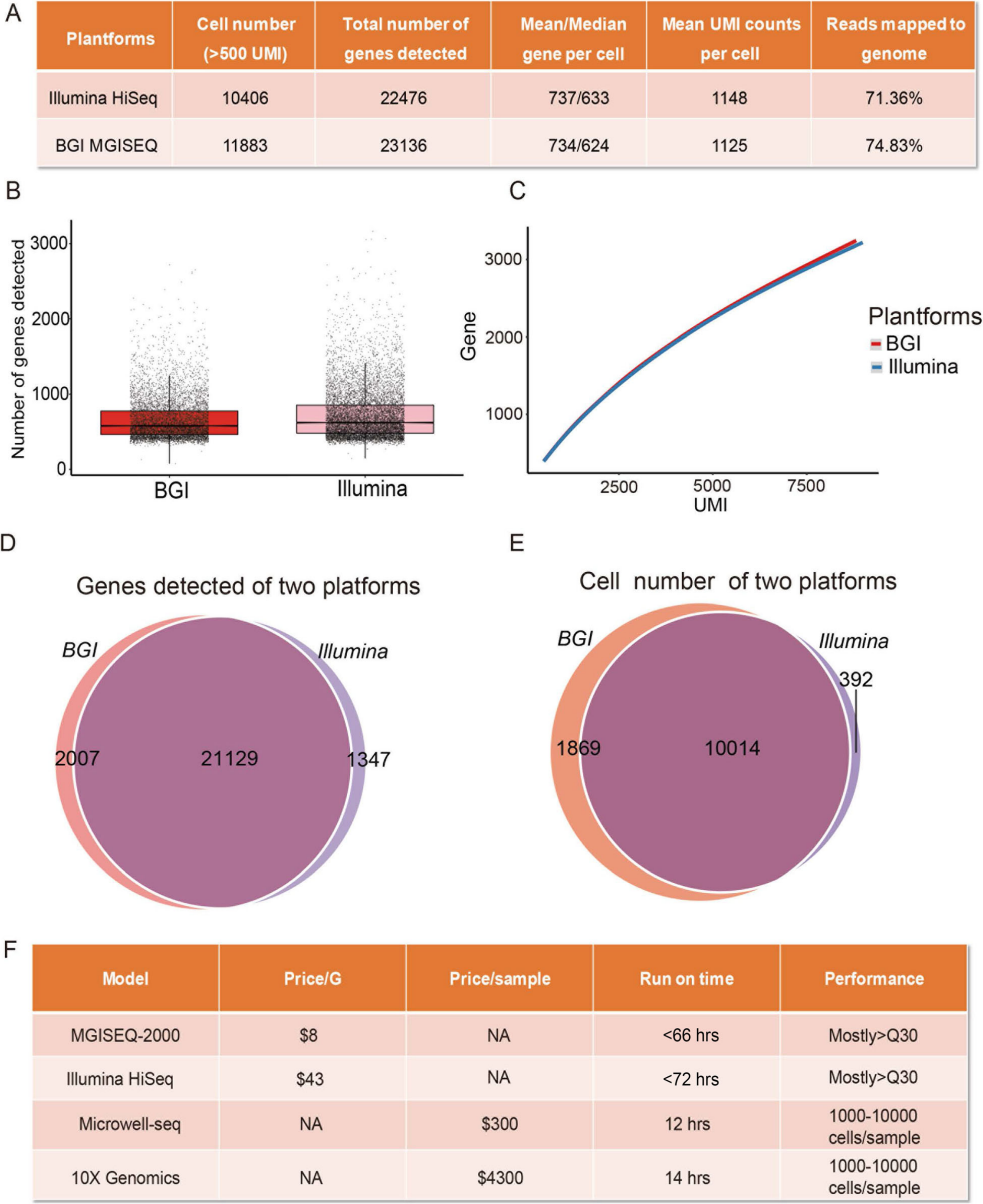
Comprehensive comparison of the BGI MGISEQ2000 and Illumina HiSeq platforms (**a**) We used BGI MGISEQ2000 and Illumina HiSeq sequencing platforms with a matched mouse adrenal gland single cell cDNA library. The BGI MGISEQ2000 and Illumina HiSeq platforms were compared in terms of cell number, total number of genes detected, mean gene number per cell, mean UMI counts per cell, and ratio of the reads mapped to the genome. **b** A bar chart of the detected gene number based on matched reads from the two sequencing platforms. **c** A line graph shows the gene number of the two sequencing platforms with respect to the matched UMIs. **d** The circle diagram summarizes the genes detected by the two platforms. We detected 23,136 genes and 22,476 genes from the sequence data generated by the BGI platform and the HiSeq platform, respectively. **e** The circle diagram summarizes the cell numbers detected by the two platforms. We detected 11,883 cells and 10,406 cells from the sequence data generated by the BGI platform and the HiSeq platform, respectively. **f** Comparison of the BGI MGISEQ2000 and Illumina HiSeq platform, Microwell-seq and 10× Genomics. Q30 is a value of sequencing accuracy, with the base misidentification < 0.1% during sequencing. A high Q30 value indicates high sequencing quality

Here, we used BGI MGISEQ2000 and Illumina HiSeq sequencing platforms with a matched mouse adrenal gland single cell cDNA library (Supplementary Table 1). Notably, our results demonstrate highly comparable performances of the BGI MGISEQ2000 and Illumina HiSeq platforms in sequencing quality, cell number, mean UMI counts per cell, median number of genes detected in an individual cell and so on (Fig. 3a-e). We detected 11,883 cells and 10,406 cells from the sequence data generated by the BGI platform and the HiSeq platform, respectively. In addition, BGI MGISEQ2000 provided more genes than the Illumina HiSeq platform when united single-cell data were used.

Mouse adult adrenal gland cells were also divided into 18 transcriptionally distinct cell types after Illumina HiSeq sequencing, using the same pipeline and parameters as were used for the previous BGI sequencing platform analysis (Supplementary Figure 4A-D). The defined cell type clusters and cluster-specific markers are summarized (Supplementary Figure 4E and Supplementary Tables 2–3). It is worth noting that the final libraries had similar clusters and gene expression patterns across the two different sequencing platforms. One key difference between the two sequencing platforms was the cost of the sequencing, which was adjusted to institutional pricing and geographical position. The cost per Gb with the MGISEQ2000 was approximately 25% that of the Illumina HiSeq sequencing platform in China (Fig. 3f). Moreover, the combination of Microwell-seq and BGI made the cost of the single-cell transcriptome analysis ~ 10 times less expensive than the currently used combination of 10× Genomics and Illumina HiSeq platforms (Fig. 3f). The cost-effectiveness and work stability make BGI MGISEQ2000 and Microwell-seq an attractive alternative.

### Cross-species comparison of human and mouse adrenal glands

High-throughput scRNA-seq technologies offer an exciting opportunity for cross-species analysis of cell types. Using Microwell-seq, we performed a comparative transcriptomic analysis of the hematopoietic system of humans and mice and revealed evolutionary conservation in the hematopoietic hierarchy across these species (Lai et al., 2018).

Here, we constructed a single-cell resolution transcriptomic atlas of the adrenal glands in mice and humans, with approximately 20,000 single cells (mice 11,883 and human 8814). The human adrenal gland was divided into 15 cell clusters that exhibited highly specific gene expression patterns (Supplementary Table 4 and Supplementary Figure 5). To obtain a detailed view of the cellular evolution from mouse to human adrenal gland tissue, we first renamed mouse genes as human orthologous genes. We divided cell types into large clusters, including immune cells, proliferating cells, stromal cells, neurons, endothelial cells, muscle cell clusters and so on (Supplementary Table 5).

**Fig. 4:**
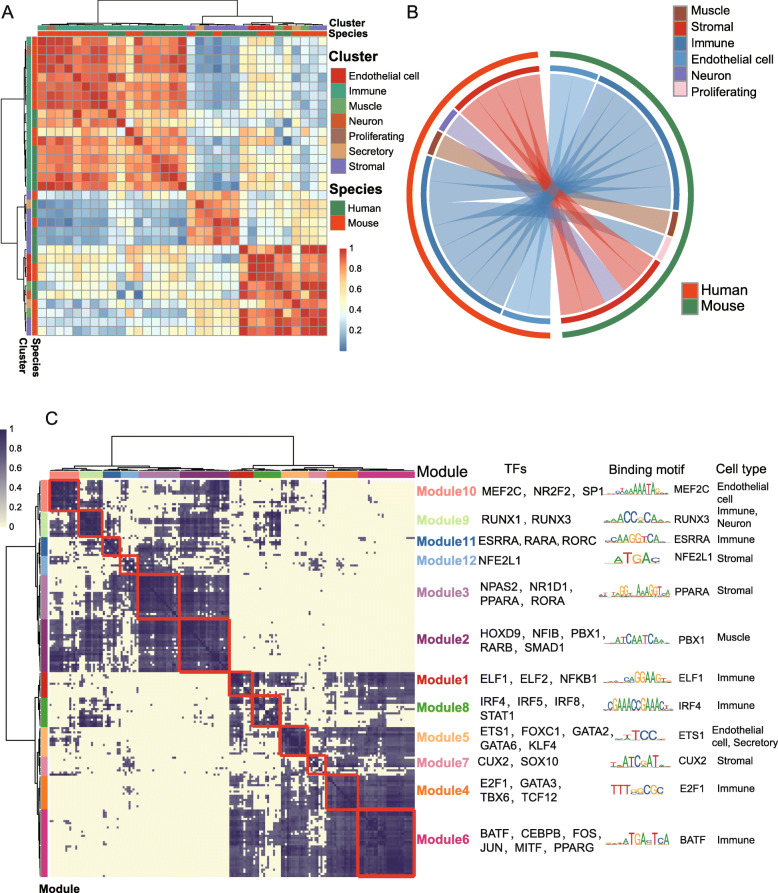
Cross-species comparisons of the adult adrenal glands (**a**) Cross-species correlation analysis of the adult adrenal glands. The fraction of cells in each human cluster was assigned to each mouse cluster based on orthologous genes. The color bar on the right represents the Z-score. Red indicates high correlation; blue and yellow indicate low correlation. **b** Circos plot showing the similarity of the cell types in adult adrenal glands of humans and mice. The human cluster is marked in red, while the mouse cluster is marked in blue, and different clusters are labeled with different colors. Paired cell types with average AUROC scores greater than 0.9 are connected by lines. **c** Identification of regulon modules module 1-module 12. This analysis was based on the regulon matrix of the human adrenal gland single-cell data and the mouse adrenal gland single-cell data. The gene regulatory network shows transcription factors (TFs) in different modules and the relationship among 12 regulon modules, along with representative TFs and the associated cell types

Moreover, the fraction of cells in each mouse adrenal gland cluster was assigned to the corresponding human adrenal gland cluster based on orthologous genes, and the correlation level was exhibited in a heat map (Fig. 4a). As shown in the heat map, the mouse muscle cluster shows strong correlations to the human muscle cluster, and the mouse stromal cluster performances were higher than those of the human stromal cluster. Then, according to the gene expression patterns of the major large cell clusters in the humans and mice, we mapped a circos plot (Gu et al., 2014). The circos plot exhibited the same correlation among clusters as shown in the heat map (Fig. 4b). It is worth mentioning that we identified four similar cell cluster pairs among humans and mice that were identical to those found with an analysis of the heat map. The mammalian cell clusters, including muscle cells, stromal cells, immune cells and endothelial cells, exhibited high correlations across both humans and mice. In addition, mouse proliferating cell performances were higher than those of human immune cells, and human neuron performances were higher than those of human stromal cells.

### Gene regulatory network comparison in these two species

Recent advances in bioinformatics methods for scRNA-seq have provided new and exciting biological insights, including the development of inferred coexpression networks and the cell state definitions (Guo et al., 2015; Moignard et al., 2015; Stegle et al., 2015; Kharchenko et al., 2014; Raj & van Oudenaarden, 2008; Trapnell et al., 2014). Cell state identification includes transcription factors (TFs) and related downstream genes, which constitute a complex and interactive gene regulatory network. The emergence of computational methods for gene regulatory network reconstruction from scRNA-seq data allows us to compare gene regulatory networks and assess the similarities and differences in TF regulation across species (Gu et al., 2014; Aibar et al., 2017; Crow et al., 2018; Hie et al., 2019; Celniker et al., 2009).

In the single-cell transcriptome data from the adrenal glands of humans and mice, the genes in the mouse regulons were first converted into homologous human genes using BioMart, and then, the orthologous TF expression was analyzed. Notably, 12 homologous TF-TF modulator modules that are highly conserved across species and participated in different signaling pathways were identified (Fig. 4c and Supplementary Figure 6). For instance, endothelial cells strongly driven by TFs such as MEF2C, NR2F2 and SP1 had the same recognition motif as the TFs in mouse (module 10). The muscle cells presented a set of conserved TFs, including HOXD9, NFIB, PBX1, RARB and SMAD1 (module 2). In addition, the stromal cells had significantly enriched motifs for the corresponding TFs, such as NPAS2, NR1D1, PPARA and RORA (module 3). Moreover, all the cell clusters showed species-conserved modules (Fig. 4c).

**Fig. 5:**
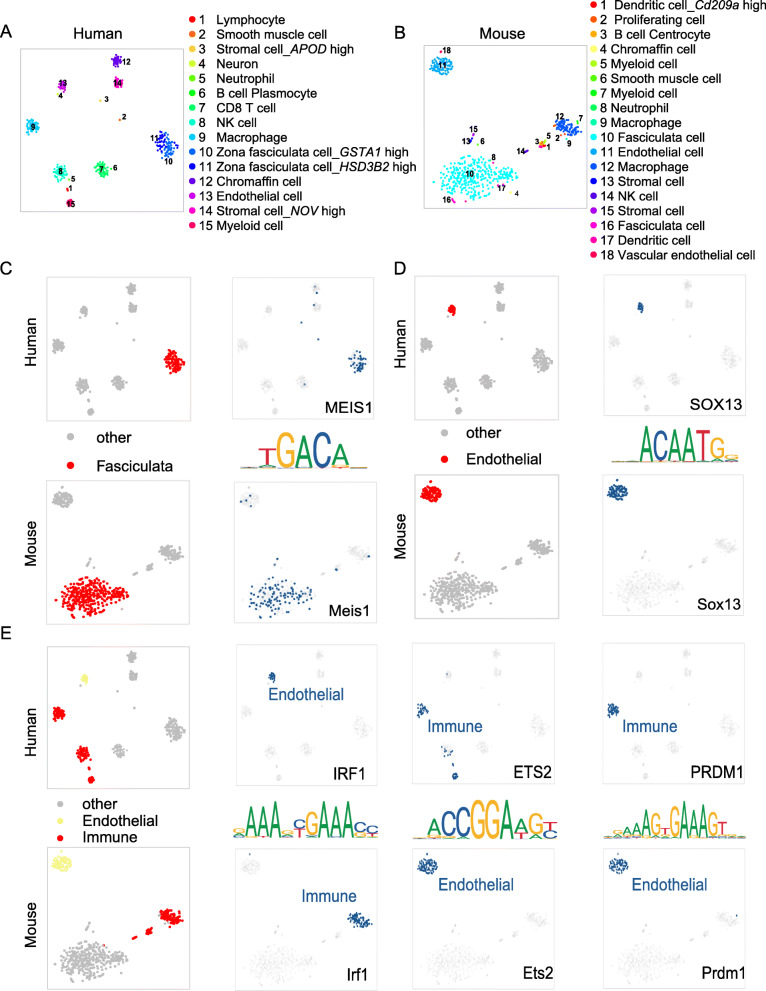
The regulon activity plot of human and mouse adrenal glands (**a**) A t-SNE map clustered by the activity of the regulons in the human adrenal gland. Cells are colored by cell-type cluster. **b** A t-SNE map clustered by the activity level of regulons in the mouse adrenal gland. Cells are colored by cell-type cluster. **c** Binarized regulon activity scores for the common human and mouse regulon MEIS1/Meis1 in the fasciculate cluster. Dark blue dots represent 1, and gray dots represent 0 in the right t-SNE map. **d** Binarized regulon activity scores for the common human and mouse regulon SOX13/Sox13 in the endothelial cluster. Dark blue dots represent 1, and gray dots represent 0 in the right t-SNE map. **e** Binarized regulon activity scores for human and mouse common regulons, including IRF1/Irf1, ETS2/Ets2 and PRDM1/Prdm1, in the endothelial or immune cell clusters. Dark blue dots represent 1, and gray dots represent 0 in the right t-SNE map

To demonstrate the conservation of TFs in humans and mice at the same gene level depth without the effects of outliers and noise, we first used pseudo-cell analysis (Materials and Methods). Then, we mapped the t-SNE map clustered by the activity of regulons (Fig. 5a-b). Each point in this t-SNE map represented an average of 20 cells randomly selected from the same cell type. In addition, we selected several regulons from fasciculata cells, endothelial cells, and immune cells for a comparison of the conservatism in the human and mouse glands. The findings showed that orthologous TFs such as MEIS1 and SOX13 regulated the same cell type in both species (Fig. 5c-d). For instance, TF MEIS1 regulated human fasciculata cells, and TF Meis1 regulated mouse fasciculata cells. On the other hand, the regulatory networks also showed some differences in the humans and mice. Specifically, TF IRF1 regulated human endothelial cells, and the TF Irf1 ortholog regulated mouse immune cells. In addition, TF IRF1 regulated human immune cells, and the TF Irf1 ortholog regulated mouse endothelial cells (Fig. 5e).

Through the GO analysis, we found different signaling pathways for the 12 homologous TF-TF modulator modules (Yu et al., 2012). For example, the module 2 cluster genes are involved in striated muscle tissue development and organ development related to muscle. In addition, the module 3 cluster genes are involved in rhythmic processes and steroid hormone-mediated signaling pathways corresponding to stromal cells (Supplementary Figure 6). We found that all 18 cell clusters in the mice showed species-conserved modules (Supplementary Table 6). In summary, our study provided 12 orthologous TF-TF regulon modules that underlie mammalian cell type conservation.

## Discussion

The novel combinations of Microwell-seq and BGI MGISEQ2000 used in our study hold many advantages over other related technologies (Macosko et al., 2015; Klein et al., 2015; Gierahn et al., 2017; Fan et al., 2015; Yuan & Sims, 2016). Microwell-seq demonstrates superiority in terms of cost, feasibility and convenience. The cost per experiment was no greater than the costs of the agarose and reagents, which were negligible. In addition, because the experimental devices require no additional equipment in addition to an ordinary microscope, it was very easy to scale up the experiment by handling multiple microwells at the same time (Lai et al., 2017). The BGI MGISEQ2000 showed high performance scRNA-seq and had competitive advantages in terms of yield, price and efficiency. The cost per Gb of the MGISEQ2000 platform was approximately 25% that of the Illumina HiSeq sequencing platform in China. Thus, the cost-effectiveness and work stability make BGI MGISEQ2000 and Microwell-seq attractive, portable, efficient, trustworthy, and inexpensive high-throughput scRNA-seq methods.

Using Microwell-seq, we presented an atlas of adult adrenal glands for mice and humans (Lai et al., 2017). Through the transcriptomic analysis of approximately 10,000 single adult adrenal gland cells in mice, we found 18 clusters that included fasciculate cells, chromaffin cells, endothelial cells, macrophages, stromal cells, natural killer cells, dendritic cells, B cells, myeloid cells, smooth muscle cells, neutrophils and proliferating cells. In addition, we demonstrated that the genes distinguish each cell cluster. Compared with human adrenal gland cells, we confirmed that the mouse adrenal gland had a special chromaffin cell and an absence of neurons. According to the results of a circos plot analysis, we showed strong evidence indicating that the major mammalian cell clusters exhibited high correlation across humans and mice; for example, the mouse muscle cluster showed strong correlation with the human muscle cluster and the mouse stromal cluster performances were higher than those of the human stromal cluster (Gu et al., 2014).

Our study also found that the regulatory networks have similarities and differences in humans and mice. It is worth mentioning that endothelial cells and immune cells in humans and mice are highly related. A variety of orthologous TFs regulate human immune cells or mouse endothelial cells, suggesting that these two cell populations may be evolutionarily related (Plass et al., 2018). Moreover, we used SCENIC to compare the gene regulatory networks in these two species (Aibar et al., 2017). In total, 12 homologous TF-TF modulator modules were confirmed in our study. These confirmed modules are highly conserved in these species and have different functional signaling pathways related to the origins of the cell types of the modules.

In conclusion, the combination of Microwell-seq and the BGI MGISEQ2000 sequencing platform opens a way for all laboratories around the world to harness the power of single-cell RNA-seq technology at little expense. We believe this system will gain popularity and benefit the biomedical research community in the near future. In addition, the adrenal gland atlas of interest will provide guidance for scientific research on adrenal glands, particularly in the clinical treatment of adrenal gland disorders and diseases, such as Cushing’s syndrome.

## Methods

### Mouse and human adrenal gland samples

Three batches of mouse adrenal glands were extracted from three adult C57BL/6 mice (6 ~ 8 weeks). The C57BL/6 mice were raised at the Experimental Animal Center of Zhejiang University. All procedures were approved by the Ethics Committee of the Experimental Animal Center of Zhejiang University. One batch of human adrenal glands was extracted from a volunteer at the First Affiliated Hospital of Zhejiang University School of Medicine. The donor signed an informed consent document. All procedures were approved by the Medical Ethics Committee of Zhejiang University Medical College.

### Mouse and human adrenal gland cell preparation

Mouse and human adrenal gland tissues were put into DMEM plus 10% FBS immediately after resection and minced using scissors into ~ 1-mm pieces on ice. The tissue pieces were transferred to a 15-ml centrifuge tube, rinsed twice with cold DPBS and suspended in 5 ml of a solution containing dissociation enzymes. The mouse adrenal glands were treated with collagen IV (37 °C for 30 min), while the human adrenal glands were treated with collagen IV (37 °C for 90 min). During dissociation, the tissue pieces were pipetted up and down gently several times until no tissue fragments were visible. The dissociated cells were centrifuged at 300×g for 5 min at 4 °C and then resuspended in 3 ml of cold DPBS. After passing through a 40-μm strainer (Biologix), the cells were washed twice, centrifuged at 300×g for 5 min at 4 °C, and resuspended at a density of 1 × 10^5^ cells/ml in cold DPBS containing 2 mM EDTA for further Microwell-seq.

### Construction of cDNA and sequencing libraries

All adrenal gland cell suspensions were treated to obtain cDNA libraries according to the standard Microwell-seq protocol (Han et al., 2018; Lai et al., 2017). Purified cDNA libraries were fragmented by a customized transposase (TruePrep DNA Library Prep Kit V2 for Illumina, Vazyme, cat #TD513), which carries two identical insertion sequences. Libraries were constructed for the MGISEQ2000 sequencing platform by a MGIEasy universal library conversion kit (App-A) (MGI, cat #1000004155). Detailed information can be found in Supplementary Table 1.

### Microwell-seq data analysis method

RNA-seq data preprocessing, alignment, quality filtering and acquisition of expression matrix cellular barcodes and unique molecular indices were achieved in read 1. We discarded the read pairs within which the base quality of any one base of the barcode was below 10, since sequencing errors and low quality may produce spurious barcodes. After tagging the cellular barcode and molecular barcode, we excluded read 1 and only used read 2 as input for the alignment. We used STAR-2.5.1b with default parameters for mapping (Dobin et al., 2013). For the UMI calculation in the case of spurious UMIs, molecular barcodes that had one base distance were merged into one. Unqualified cells, those with fewer than 500 transcripts, were excluded. Cells with a high proportion of transcripts derived from mitochondria-encoded genes were also excluded.

### Clustering of mouse adrenal gland cells and comparing data generated by different sequencing platforms

We used Seurat software (Satija et al., 2015) for clustering. The digital gene expression (DGE) data were used for the input. We considered the batch effect and library size to be the main sources of unwanted variation and used a linear model regression to eliminate unwanted variations of variables before the PCA analysis. This approach fit the batch effect well. The cells from 3 batches were well mixed on the t-SNE map, but separation by physical differences was not regressed out of the data set. PCA analysis was performed, and the top 100 principal components were contained (Satija et al., 2015). To compare mouse adrenal gland data produced by BGI MGISEQ2000 and Illumina HiSeq, we classified cells using the same sequencing depth and analysis principles. The heat map produced by the DoHeatmap function was one of the bases for judging the quality of the clustering. Highly differentially expressed genes are represented on a heat map. A more detailed description of the data analysis can be found on the following website: github.com/ggjlab/mca_data_analysis.

### Cross-species comparison of the adrenal gland transcriptome

To make the cross-species transcriptome comparable, we used the homology correspondence values from dmodENCODE (Celniker et al., 2009). Both gene expression profiles were normalized to the total number of transcripts and multiplied by 100,000. We used pseudo-cells for further analysis to attenuate the effects of outliers and noise. Each pseudo-cell represented an average of 20 cells randomly selected from the same cell type. To compare cross-species transcriptomes, we performed MetaNeighbor (Crow et al., 2018) analysis between humans and mice. MetaNeighbor quantified cell type replicability using neighbor voting. The mean area under the receiver operator characteristic curve (AUROC) scores was used to measure the similarity of cell types, and we chose 0.9 as the threshold. We used the circlize (Gu et al., 2014) package and the pheatmap package to visualize the similarity of the cell types of the humans and mice.

### Cross-species regulatory network comparison of the adrenal glands

We used SCENIC (Aibar et al., 2017) to compare the gene regulatory networks across species. We combined the AUCell score matrices with the orthologous TF. The TF modules were identified based on the connection specificity index (CSI) (Fuxman Bass et al., 2013). The cross-species regulatory networks were constructed based on the TF modules and visualized with a heat map. To demonstrate the functions enriched in each module, we performed GO analysis for these modules.

### Cross-species regulon activity analysis

AUCell provided the activity of the regulons across the cells. To see whether there are conserved regulons active in specific cell types in the humans and mice, we created the “binarized regulon activity matrix”, with the coordinates of the matrix that correspond to active regulons in a given pseudocell containing a “1” value, and “0” is attributed to all the others. Then, we used the SCENIC R package (Aibar et al., 2017) to run the t-SNEs for both data sets and clustered the cells based on this regulon activity. The regulon activity can be shown on a t-SNE map by setting artificial thresholds.

## Supplementary information

**Additional file 1: Figure S1.** Representative gene expression in the mouse adrenal glands. **Figure S2.** The different expression patterns of the two stromal cell clusters. **Figure S3.** The different expression patterns of the two myeloid cell clusters. **Figure S4.** Comparison of BGI MGISEQ2000 and Illumina HiSeq platforms based on the t-SNE and heat maps. **Figure S5.** Mapping the human adult adrenal gland atlas by Microwell-seq. **Figure S6.** The signaling pathways of several representative TF modules.

**Additional file 2: Table S1.** Detailed information on the seven sequencing data sets.

**Additional file 3: Table S2.** Markers of mouse adrenal gland cells by a BGI platform.

**Additional file 4: Table S3.** Markers of mouse adrenal gland cells by an Illumina platform.

**Additional file 5: Table S4.** Differentially expressed genes detected in the human adrenal gland.

**Additional file 6: Table S5.** A list of redefined large cell clusters.

**Additional file 7: Table S6.** Human-mouse 12 TF modules.

## Data Availability

The accession numbers for the raw data files used for the RNA sequencing analysis reported in this paper are GSE108097 and GSE134355.
