# Supplementary figures and images for: Mapping a mammalian adult adrenal gland hierarchy across species by microwell-seq
Source: Cell Regen. 2020 Aug 3;9:11. doi: 10.1186/s13619-020-00042-8 (PMC7396412; doi:10.1186/s13619-020-00042-8)

Figure S1

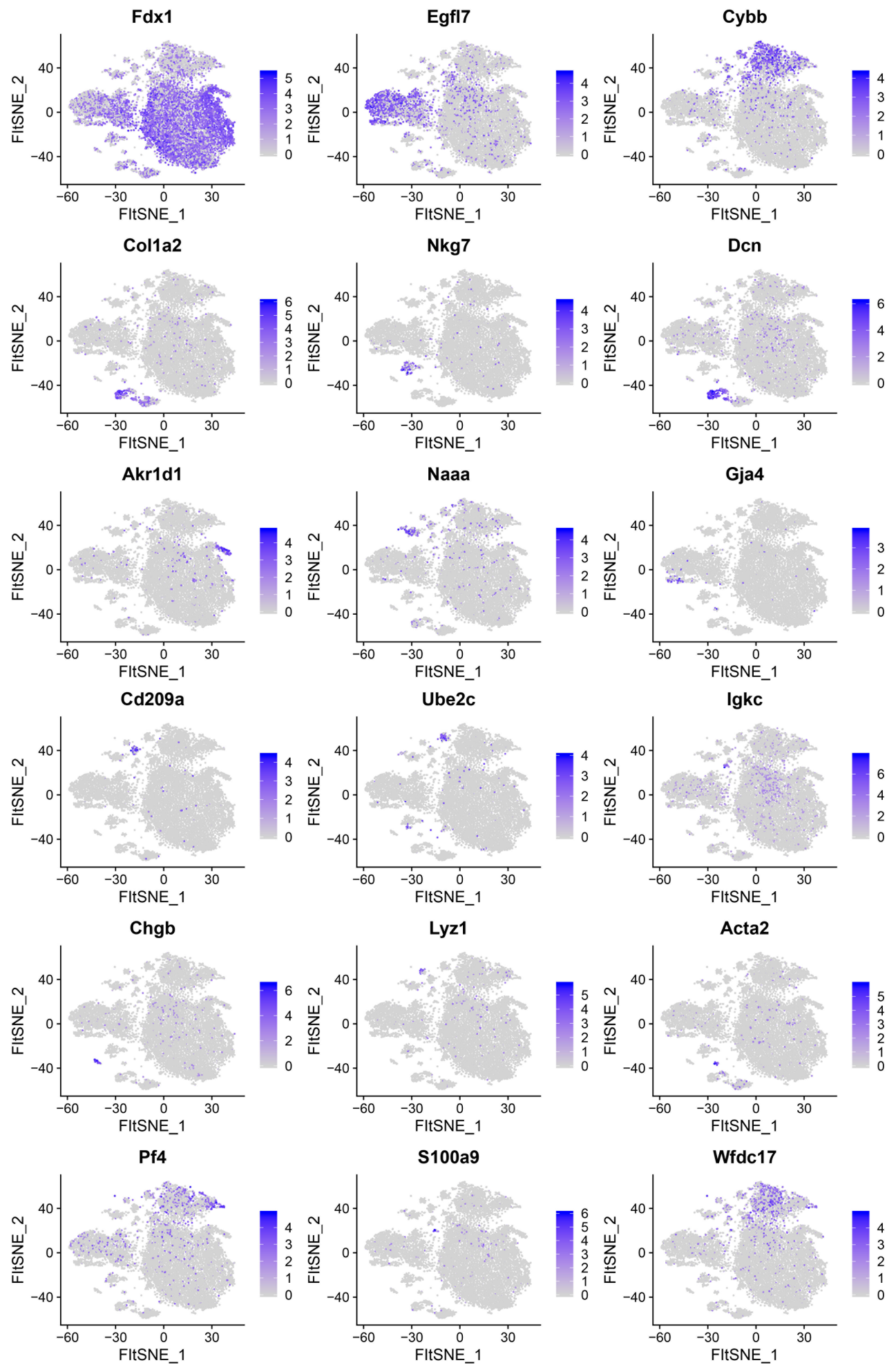

Figure S2

A

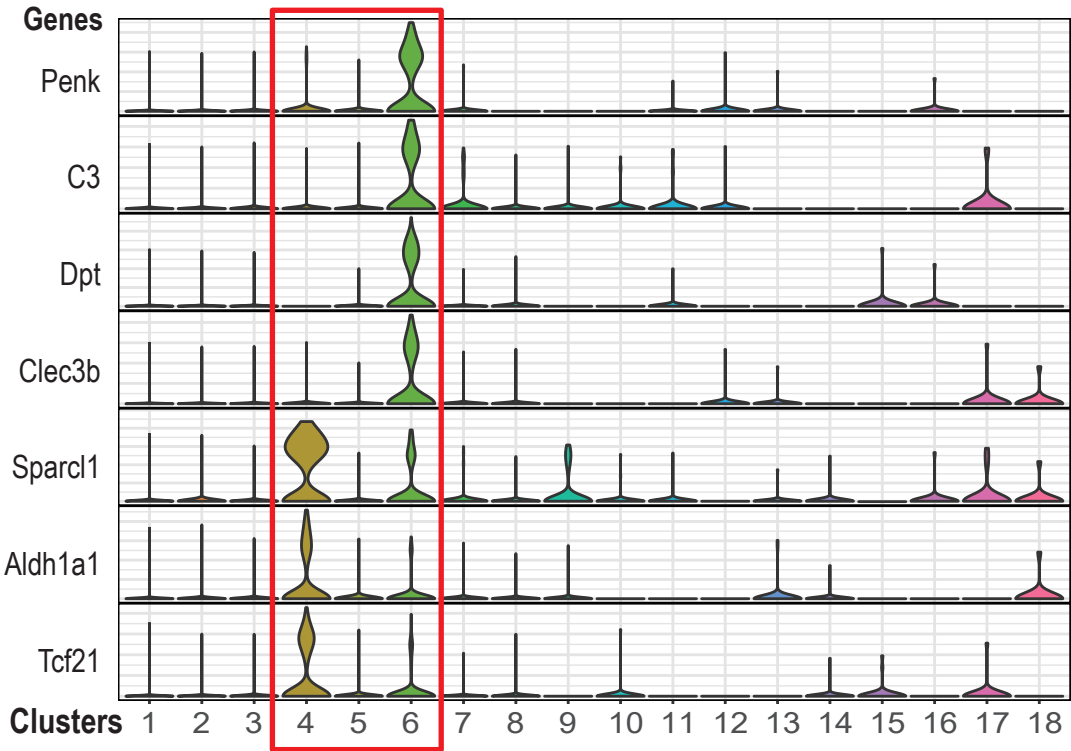

B

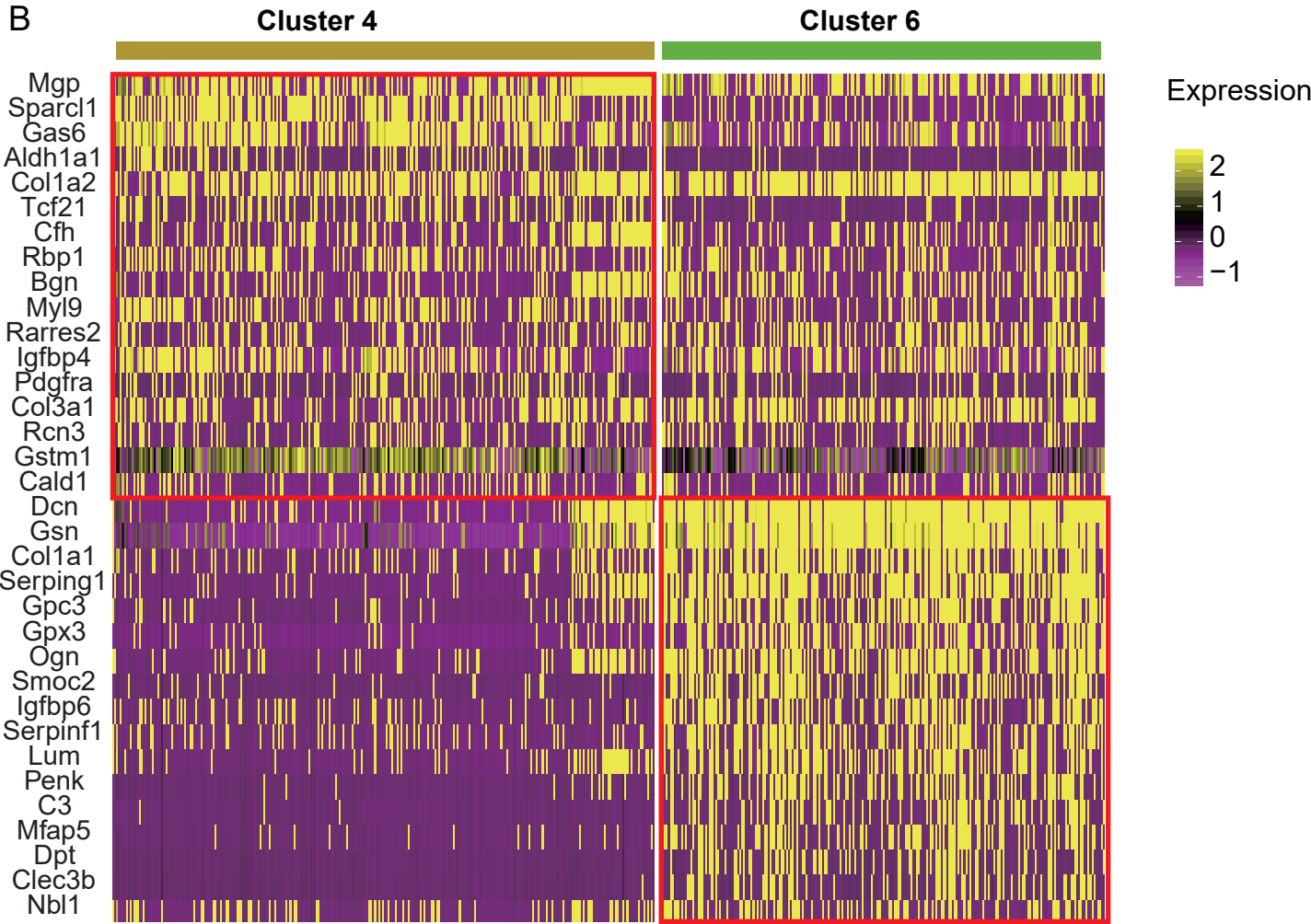

Figure S3

A

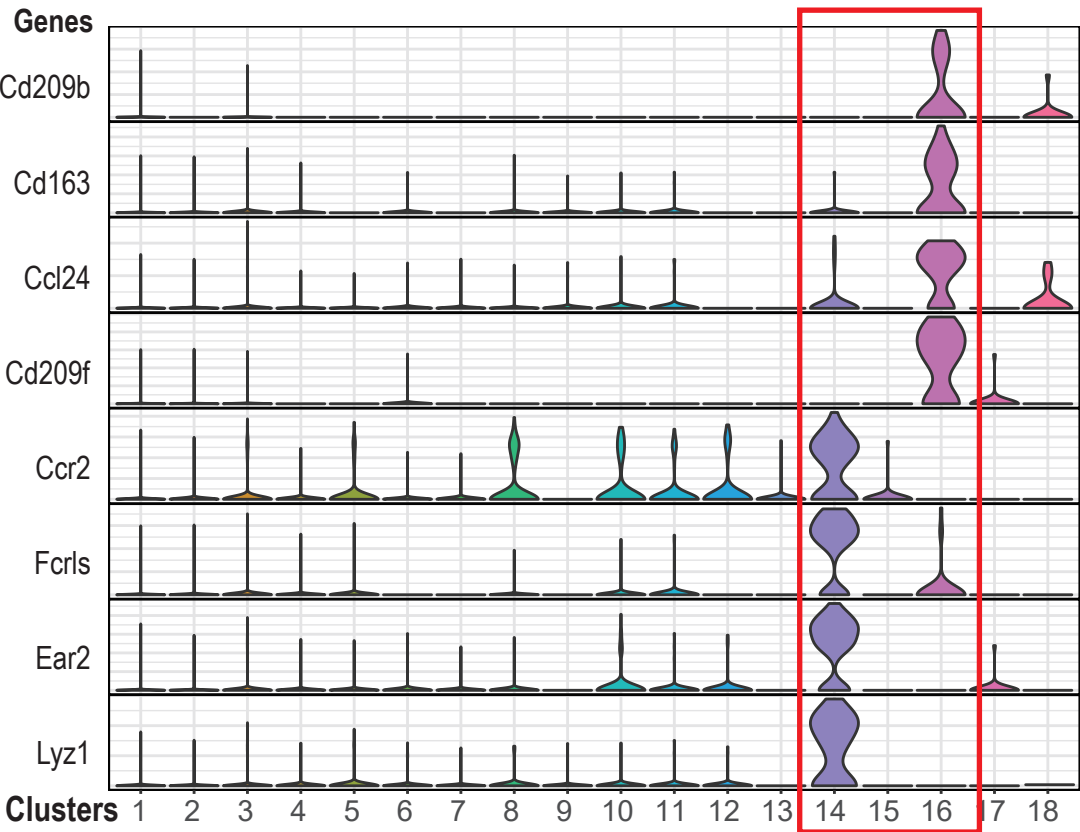

B

Cluster 14

Cluster 16

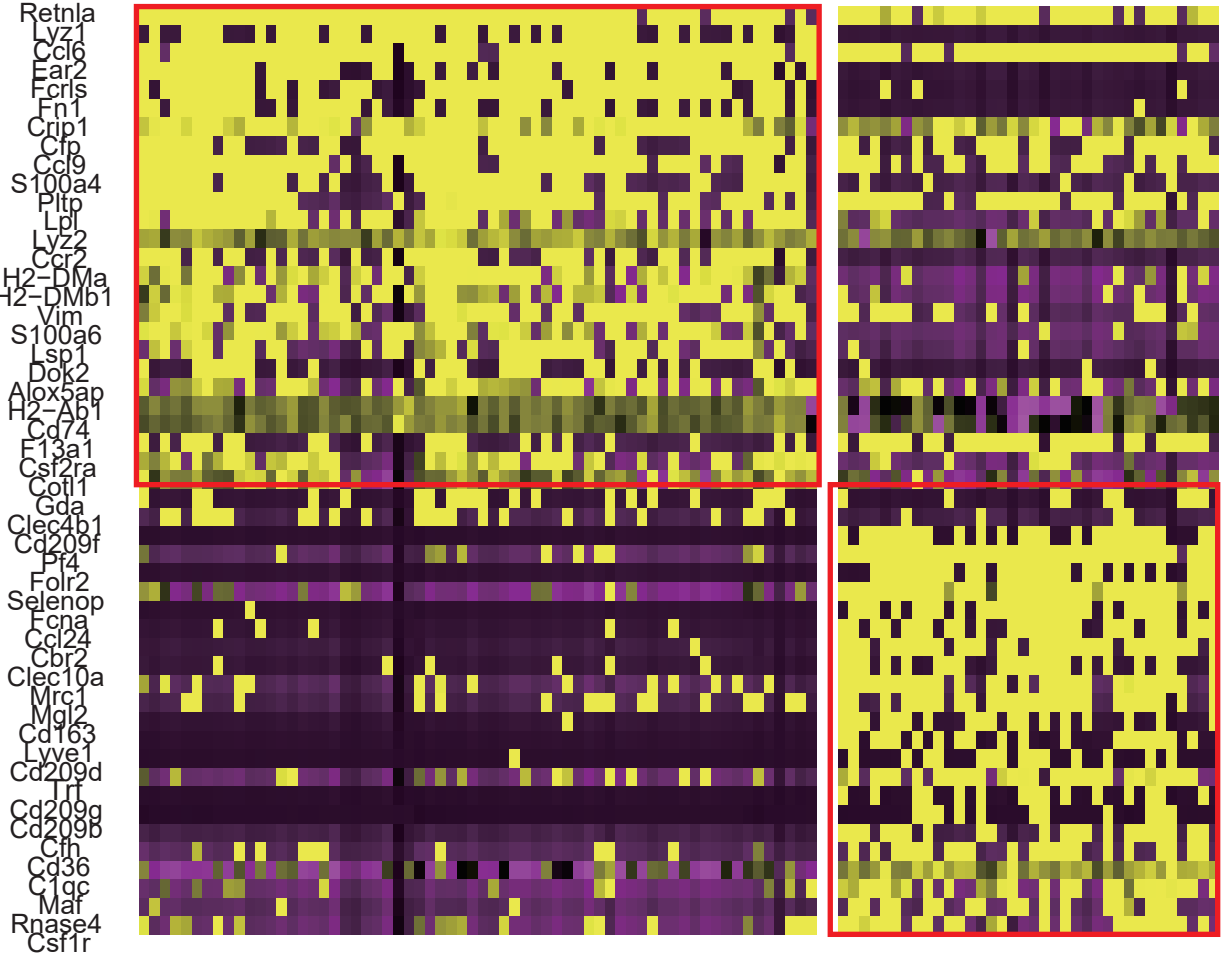

Figure S4

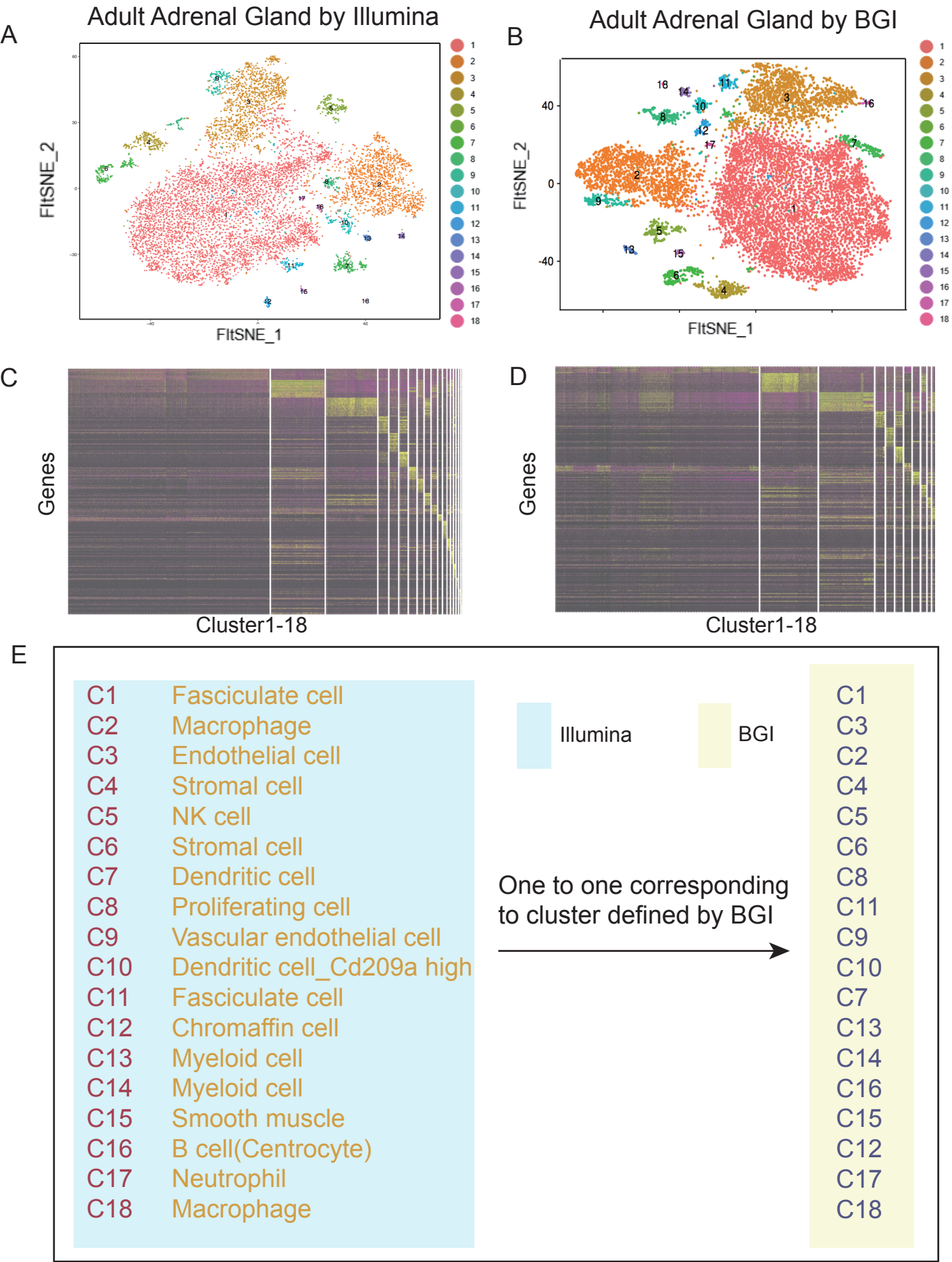

Figure S5

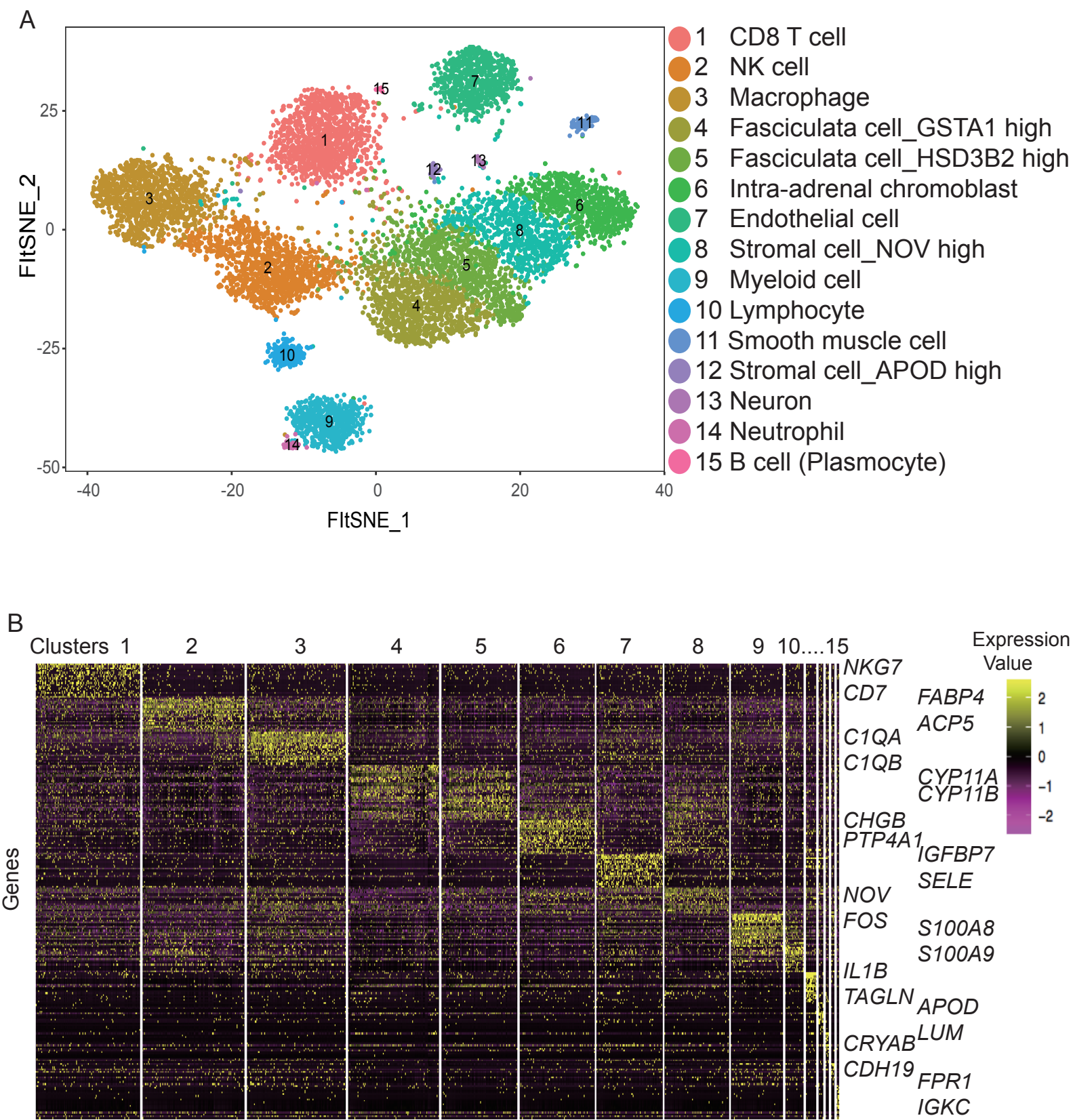

Figure S6

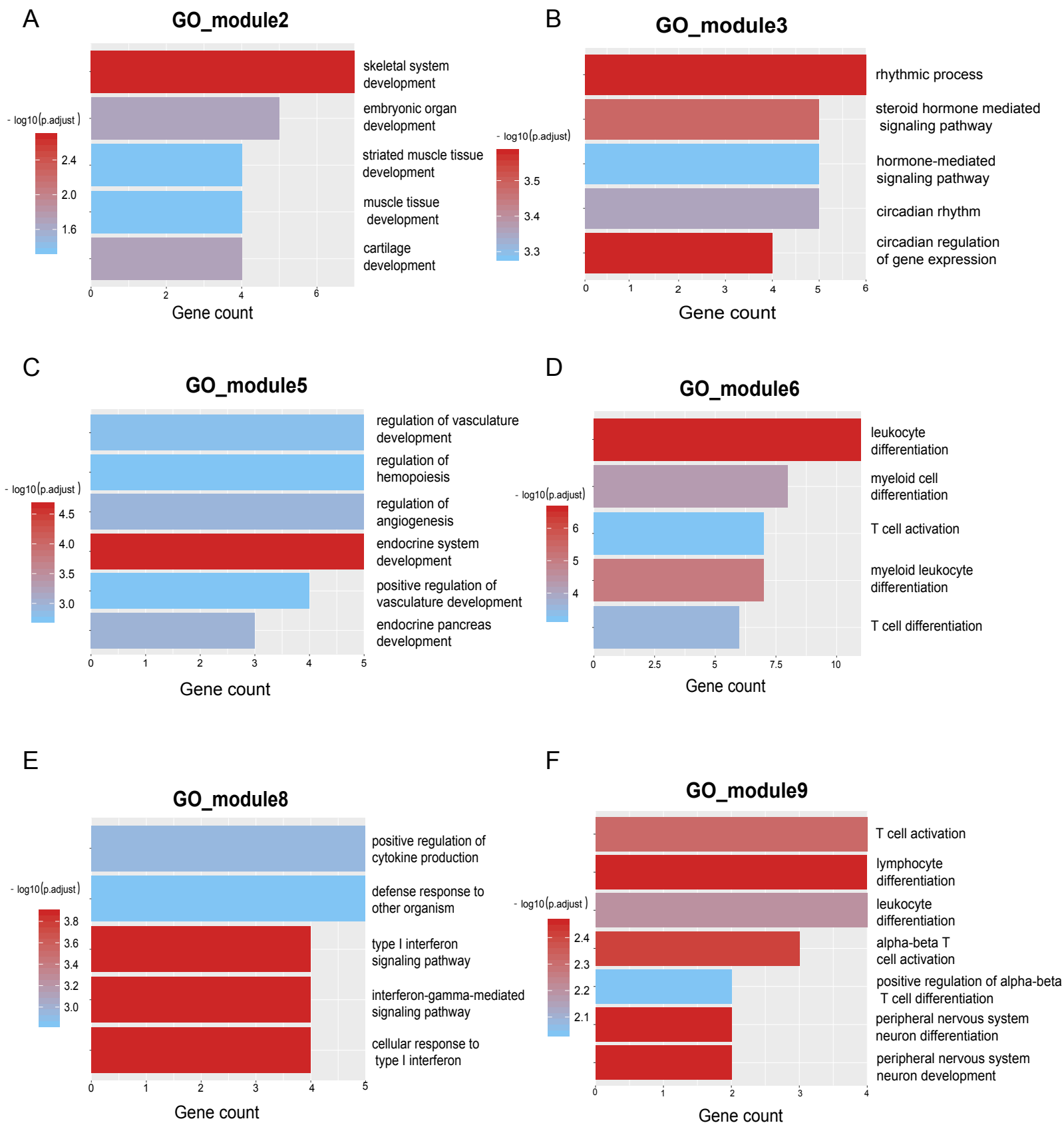

Supplement: Supplementary file 1 — Additional file 1: Figure S1. Representative gene expression in the mouse adrenal glands. Figure S2. The different expression patterns of the two stromal cell clusters. Figure S3. The different expression patterns of the two myeloid cell clusters. Figure S4. Comparison of BGI MGISEQ2000 and Illumina HiSeq platforms based on the t-SNE and heat maps. Figure S5. Mapping the human adult adrenal gland atlas by Microwell-seq. Figure S6. The signaling pathways of several representative TF modules. [file 13619_2020_42_MOESM1_ESM.pdf]
